# Urothelial carcinoma of the prostate with raised β-hCG levels: a case report

**DOI:** 10.1186/s13256-022-03458-9

**Published:** 2022-06-15

**Authors:** Julia Sołek, Marta Kalwas, Magdalena Sobczak, Sylwia Dębska-Szmich, Piotr Kupnicki, Dorota Jesionek-Kupnicka

**Affiliations:** 1 Department of Pathology, Chair of Oncology, Medical University of Lodz, ul. Pomorska 251, 90-001 Lodz, Poland; 2 Department of Chemotherapy, Chair of Oncology, Medical University of Lodz, ul. Paderewskiego 4, 93-509 Lodz, Poland; 3 Department of Radiology and Diagnostic Imaging, Medical University of Lodz, ul. Paderewskiego 4, 93-509 Lodz, Poland

**Keywords:** Urothelial cancer, β-hCG, Prostate cancer, Case study

## Abstract

**Background:**

Trophoblastic differentiation in primary urothelial carcinoma of the prostate is extremely rare. An increased level of β-subunit human chorionic gonadotropin in serum in urothelial carcinoma is detected in approximately 30% of cases. To our knowledge, increased concentration of β-subunit human chorionic gonadotropin in serum in prostatic urothelial carcinoma has never been reported and its clinical significance is not evaluated yet.

**Case report:**

Here we present the case of a 67-year-old European patient who was admitted to the hospital with hematuria, dysuria, and enlarged painful testis. Ultrasonographic examination of the testis did not reveal any focal lesion. Magnetic resonance imaging of the pelvis showed a tumor of 62 mm diameter mainly located in the posterior part of the prostatic gland. A pathological examination from cystoscopy biopsy allowed us to set the diagnosis of high-grade invasive urothelial carcinoma with trophoblastic differentiation. The patient received neoadjuvant treatment. Nonetheless, after a short period of disease stabilization, he developed progression and brain metastasis. He died 9 months after diagnosis. During the disease course, his β-human chorionic gonadotropin level was measured repeatedly and analyzed in relation to disease progression. The level of serum β-human chorionic gonadotropin corresponded with the therapy response; it was at its lowest during stabilization and the highest in the metastatic stage.

**Conclusion:**

Our case study provides the first report of urothelial cancer of the prostate, with a concomitant increase of β-subunit human chorionic gonadotropin level with testis enlargement. Besides its rarity, it constitutes an interesting observation of increasing β-subunit human chorionic gonadotropin concentration with concomitant disease progression.

## Background

Primary urothelial carcinoma of the prostate is a rare malignancy (incidence 1–5%) with an aggressive course and poor prognosis [1]. Overall survival does not exceed 2 years as it tends to recur and metastasize quickly [1]. As trophoblastic transformation in prostatic urothelial carcinoma is extremely rare, the prognostic significance of β-subunit human chorionic gonadotropin (β-hCG) has not been investigated yet [2]. Trophoblastic differentiation results in the presence of syncytiotrophoblastic cells and in some cases in increased β-hCG levels in serum [3]. The presence of β-hCG in serum or urine has been already reported in many other malignancies, including lung, breast, stomach, bladder, and prostate adenocarcinoma [4–8]. It is perceived as a marker of progression as it promotes growth and invasion of tumor cells while correlating with high-grade and advanced-stage disease [9–12]. Owing to insufficient evidence of β-hCG utility in urothelial cancer, it is nowadays not widely used in clinical practice [12]. Here we present the case of a 67-year-old male patient with urothelial carcinoma of the prostate, non-neoplastic testis enlargement, and increased concentration of β-hCG with fluctuations during the course of the disease.

## Case presentation

**Fig. 1:**
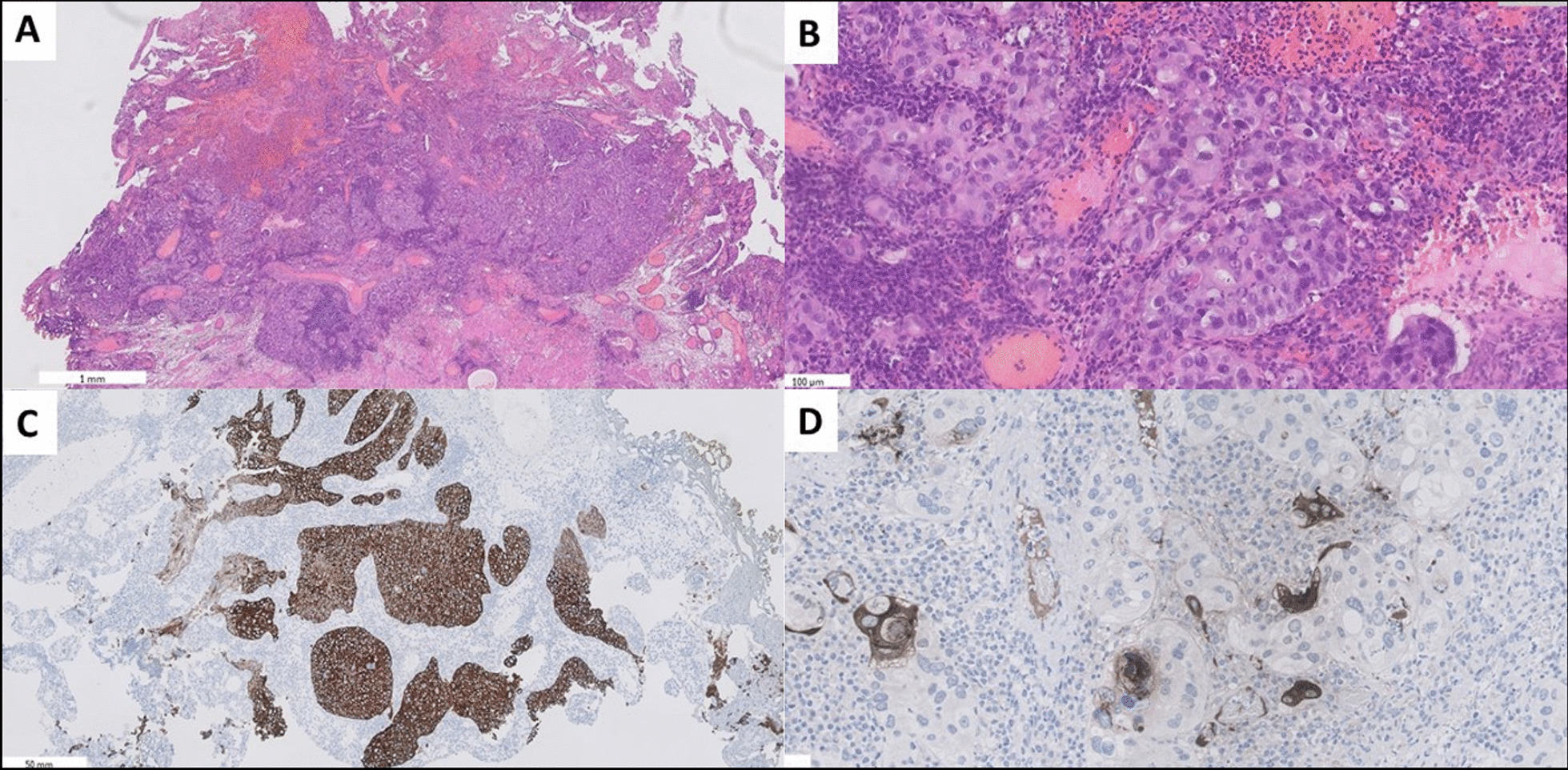
Histopathological image of specimen from cystoscopy. **A** Gross picture of invasive high-grade urothelial carcinoma [hematoxylin and eosin staining (H&E); 10× magnification]. **B** Syncytiotrophoblastic cells in the urothelial carcinoma (H&E; 400× magnification). **C** Positive immunohistochemical (IHC) staining for cytokeratin 20 (CK20, DAKO) in nest of carcinoma cells (50× magnification). **D** Positive IHC reaction for β-human chorionic gonadotropin in large syncytiotrophoblastic cells inside the urothelial carcinoma (anti-β-hCG, DAKO, 100× magnification)

**Fig. 2:**
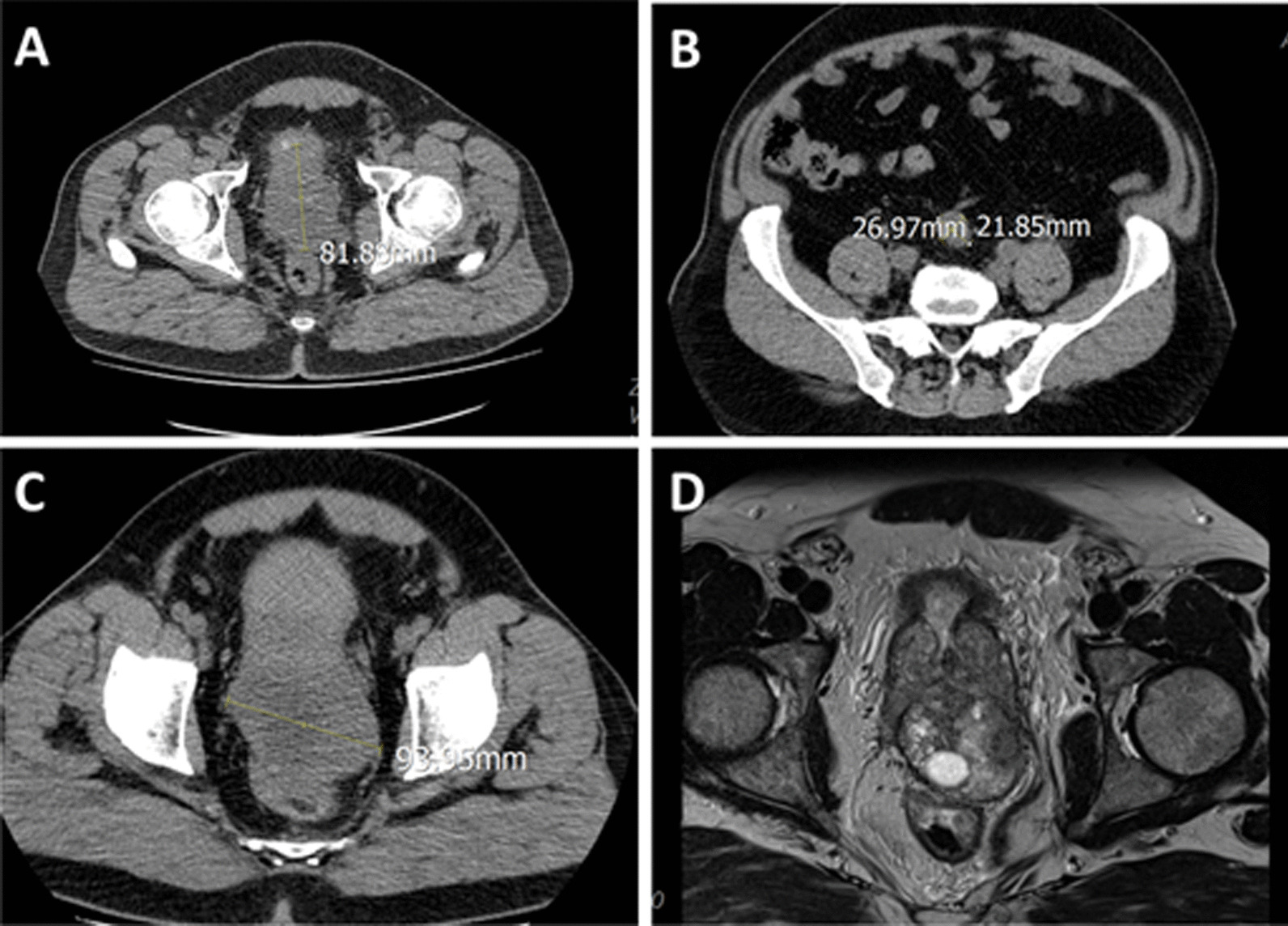
Imaging studies of patient’s urothelial tumor after progression on computed tomography (CT) (**A**–**C**) and magnetic resonance imaging (MRI) (**D**). **A** Longitudinal dimension of the tumor. **B** Short-axis diameter of pelvic lymph node. **C** Transverse dimension of tumor. **D** MRI of patient’s tumor in pelvis

A 67-year-old European man was admitted to the Department of Urology in December 2019 with major complaints regarding recurrent hematuria, dysuria, pain, and enlargement of the left testicle, pain in the lumbar and sacral section of the spine, and hyperhidrosis. The patient also reported a recent loss of weight: around 3 kg down from 97 kg within the last month. A medical interview revealed occupational exposure to asbestos since the patient had been working in a factory producing asbestos seals for several years. His body mass index was 37.8 kg/m^2^, and body surface area was 2.09 m^2^. Physical examination revealed lower limb edema as well as painful and enlarged left testis of regular shape and firm consistency, without any palpable focal lesion. Owing to enlarged testis, ultrasonography imaging (USG) and blood tests were performed. USG revealed diffuse testis enlargement but did not reveal any focal malignancies originating from the testis. Results of the blood test showed an increased level of β-hCG (644.4 lU/l). The other tumor markers, that is, lactate dehydrogenase (LDH) α-fetoprotein (AFP), and prostate-specific antigen (PSA) remained within the normal range (230 U/ml, 1.58 U/ml, and 0.874 ng/ml, respectively). Surprisingly, the following magnetic resonance imaging (MRI) revealed a mass up to 62 mm in the posterior part of the prostatic gland, infiltrating seminal vesicles and anterior rectum wall together with bilaterally enlarged iliac lymph nodes. Additionally, computed tomography (CT) confirmed metastasis in pelvic lymph nodes. Cystoscopy was performed, and a biopsy specimen from tumor mass from prostate and bladder was taken. Histopathological examination of the specimens obtained in the cystoscopy revealed prostate and muscle of the bladder infiltration consisting of high-grade invasive urothelial carcinoma with trophoblastic differentiation and the presence of large syncytiotrophoblastic cells. Urothelial carcinoma cells were positive for cytokeratin 20 (CK20), GATA3, p63, and CKHMW but negative for PSA. Some of these large syncytiotrophoblastic cells inside the tumor showed positivity for β-hCG (Fig. 1). Keeping in mind that urothelial cancer most often originates in the bladder, the patient was diagnosed with urothelial bladder cancer. However, reassessment of the radiographic imaging led to the conclusion that the tumor was predominantly located in the prostate, which suggested the prostate as the primary origin and established the diagnosis of urothelial prostate cancer with secondary bladder involvement. However, the treatment of urothelial cancer remains the same regardless of primary origin. As subsequent radiography imaging of the chest did not reveal any focal changes, the presence of metastasis in the chest was excluded. In March 2020, the patient started neoadjuvant chemotherapy with intravenous cisplatin 75 mg/m^2^ on the first day and intravenous gemcitabine 1000 mg/m^2^ on the 1st, 8th, and 15th repeating cycle every 4 weeks (cisplatin–gemcitabine protocol, PG). His symptoms improved significantly, and the β-hCG concentration decreased (205.2 mIU/ml) within the first 2 months of therapy. However, after two cycles, β-hCG level slightly increased (504.8 mIU/ml) and hyperhidrosis recurred. After four cycles, computed tomography (CT) revealed that local treatment could not be implemented owing to rectum infiltration. PG chemotherapy was continued up to six cycles. Four months later, after completion of PG chemotherapy, MRI showed disease progression. Pelvic tumor enlarged to 90 mm in diameter, and pelvic lymph nodes enlarged to maximum 21 mm in short-axis diameter (Fig. 2). Simultaneously, β-hCG concentration increased to 9446 mIU/ml. In October 2020, palliative chemotherapy was implemented (paclitaxel 80 mg/m^2^ once a week). After the fifth cycle of paclitaxel, the patient presented with polyneuropathy, so gabapentin was administered but withdrawn because of dizziness. However, the dizziness aggravated, so a CT scan of the brain was performed. It revealed small, diffuse metastases up to 8 mm in the pons. At that time, the β-hCG concentration reached the highest value of 31,163 mIU/ml. Palliative radiotherapy was planned, but owing to rapid deterioration of general condition (PS ¾), the patient did not manage to start the treatment. Eventually, the patient died in November 2020, 2 weeks after diagnosis of brain metastasis, because of disease progression.

## Discussion and conclusions

**Fig. 3:**
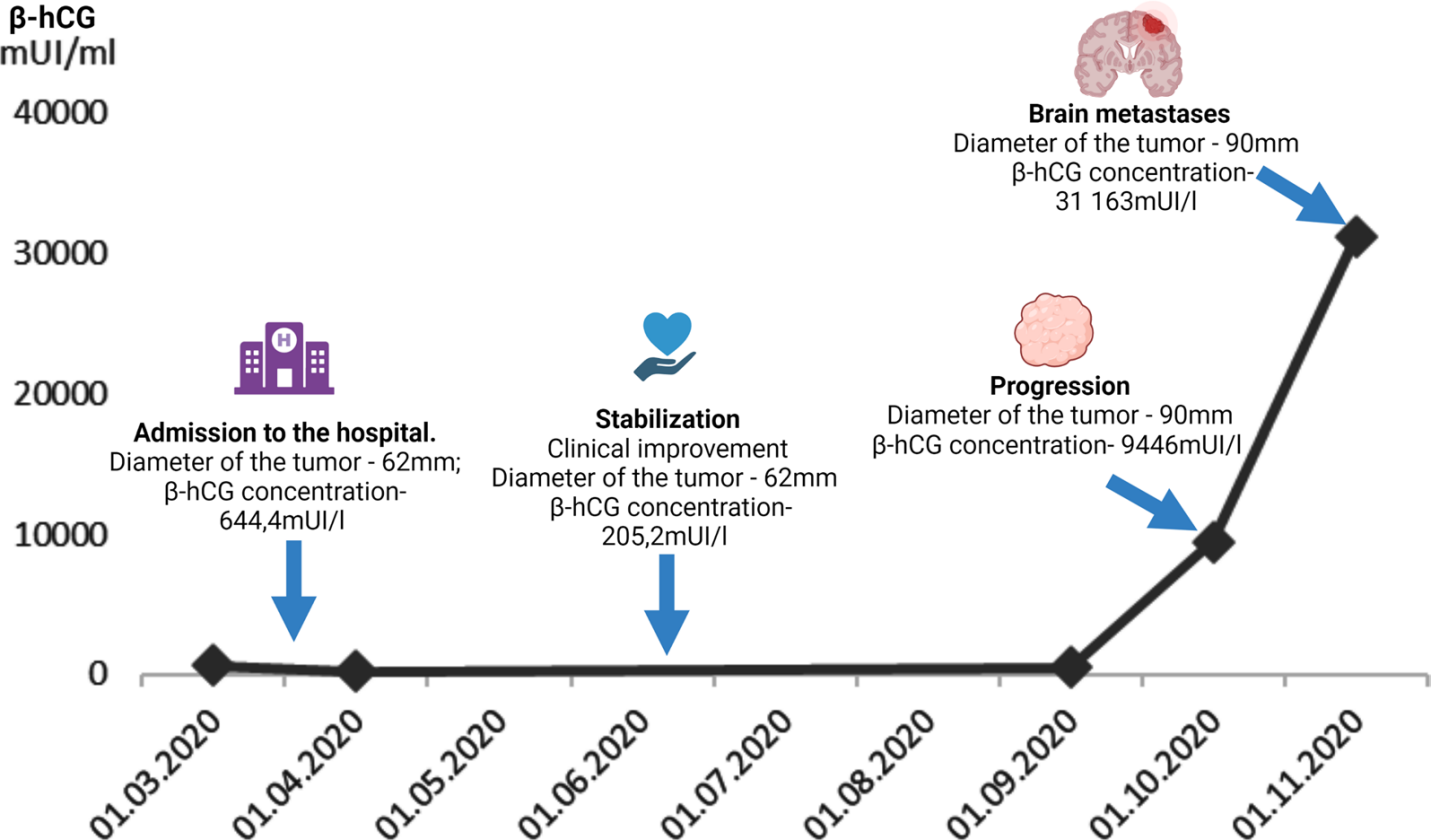
Relation between concentration of β-hCG and course of the disease

β-hCG is routinely used in testicular cancer in multiple stages of the disease. It supports diagnostics, correlates with stage and patients’ risk, and helps to monitor response to the treatment or predicts disease relapse [13, 14]. In urothelial cancer of the bladder, it showed to have a prognostic value. Douglas *et al*. utilized β-hCG serum levels in patients with urothelial transitional cell carcinoma to predict patients’ prognosis. They showed that total serum level of β-hCG is an independent prognostic factor in patients receiving chemotherapy for urothelial transitional cell carcinoma [9]. This observation was further confirmed in other studies, which showed that expression of β-hCG in bladder urothelial cancer is associated with radioresistance, high grade, and muscle invasion [15, 18, 19]. Moreover, Malkhasyan *et al*. reported a case where the level of β-hCG corresponded with tumor progression and treatment response [20]. High levels of β-hCG were shown to be associated with high grade, resistance to chemotherapy, and poor survival in other malignancies as well, such as in lung cancer and colorectal cancer [16, 17]. It should be remembered that an elevated level of β-hCG may also affect the secondary hyperplasia of organs. It is reported to influence the growth of breasts causing gynecomastia and enlargement of testicles and penis [21–23]. Here we present a unique case of testis enlargement caused by β-hCG produced by urothelial prostate cancer. Increased levels of β-hCG and testicle enlargement lead to the suspicion of a germ cell tumor malignancy, which was excluded on USG imaging. Presumably, the β-hCG elevation caused a secondary growth of the testicles, which could mislead the diagnosis. Moreover, to our knowledge, the β-hCG concentration of 31,163 mUl/ml is the highest recorded so far. Owing to multiple measurements of serum β-hCG concentration during disease, our case presents the dynamics of β-hCG level and its further association with disease progression. At the beginning of treatment, the patient presented a good response with the stabilization and decrease of β-hCG to 205.2 mIU/ml. One month after stabilization, the level increased to 504.8 mIU/ml, and 5 months later, disease progression was observed. Enlargement of the tumor to the size of 90 mm and brain metastasis resulted in the increase of β-hCG to 9446 mIU/ml and 31,163 mIU/ml, respectively. Fluctuation of β-hCG with disease progression is shown in Fig. 3.

This case report is in our opinion a valuable example of a nonobvious presentation of high-grade invasive urothelial carcinoma of the prostate with trophoblastic differentiation and increased β-hCG serum concentration and secondary testis enlargement. Moreover, repeatedly measured β-hCG provided an occasion to observe how its serum concentration correlates with gradual disease progression. To our knowledge, urothelial prostate cancer has been reported in the literature only once, but this is the first report of such clinical presentation with increased concentration of β-hCG and testis enlargement [2]. We hope that our case will lead to the widening of the clinical perception of urothelial prostate cancer and the extension of the differential diagnosis of neoplasms with a high β-hCG level to include urothelial prostate cancer with trophoblastic differentiation.

## Data Availability

Not applicable.

## Abbreviations

β-hCG: β-Subunit human chorionic gonadotropin
USG: Ultrasonography imaging
LDH: Lactate dehydrogenase
AFP: α-Fetoprotein
PSA: Prostate-specific antigen
CT: Computed tomography
MRI: Magnetic resonance imaging
PG: Cisplatin–gemcitabine protocol
